# Endothelial Cell Phenotypes are Maintained During Angiogenesis in Cultured Microvascular Networks

**DOI:** 10.1038/s41598-018-24081-z

**Published:** 2018-04-12

**Authors:** Jessica M. Motherwell, Christopher R. Anderson, Walter L. Murfee

**Affiliations:** 1 Department of Biomedical Engineering, Tulane University, New Orleans, LA 70118 United States; 2 Department of Chemical and Biomolecular Engineering, Lafayette College, Easton, PA 18042 United States; 3 J. Crayton Pruitt Family Department of Biomedical Engineering, University of Florida, Gainesville, FL 32611 United States

## Abstract

A challenge in tissue engineering biomimetic models for studying angiogenesis is building the physiological complexity of real microvascular networks. Our laboratory recently introduced the rat mesentery culture model as an *ex vivo* experimental platform for investigating multicellular dynamics involved in angiogenesis within intact microvascular networks. The objective of this study was to compare endothelial cell phenotypes along capillary sprouts in cultured *ex vivo* rat mesentery microvascular networks to *in vivo* endothelial cell phenotypes. For Day 3 (*Ex Vivo*) tissues, adult rat mesentery tissues were cultured for three days in media supplemented with 10% serum. For Day 3 (*In Vivo*) tissues, adult rats were anesthetized and the mesentery was exteriorized for twenty minutes to induce angiogenesis. Microvascular networks from Day 3 (*Ex Vivo*) and Day 3 (*In Vivo*) groups were angiogenic, characterized by an increase in vessel density, capillary sprouting, and identification of similar BrdU-positive endothelial cell distributions along sprouts. Endothelial cells in both groups extended pseudopodia at the distal edge of capillary sprouts and displayed similar endothelial cell UNC5b, VEGFR-2, and CD36 labeling patterns. The results from this study support the physiological relevance of the rat mesentery culture model and highlight its novelty as a biomimetic tool for angiogenesis research.

## Introduction

Angiogenesis, defined as the growth of new blood vessels within microvascular networks, is a complex biological process that integrates multiple cell types, intracellular and paracrine signaling, cell-cell interactions, extracellular matrix proteins, and soluble factor guidance cues^1^. This process is involved in tumor growth, myocardial infarction, diabetic retinopathy and numerous other pathologies^2,3^. Despite extensive research progress, a need still exists to better understand the specific cellular and molecular mechanisms associated with angiogenesis. The drive to advance our basic understanding of these processes and the development of therapies aimed at manipulating angiogenesis has motivated the emergence of biomimetic models. These models aim to recapitulate the cellular dynamics involved in angiogenesis using *in vitro*, *ex vivo*, and *in silico* systems so that specific interactions can be probed in ways not possible *in vivo*. Consider endothelial cell phenotypes along capillary sprouts, commonly referred to as tip cells and stalk cells^4^. Tip cells lead endothelial sprouts and are distinct from stalk cells by the presence of cytoskeletal extensions, known as pseudopodia, and their increased expression of unc-5 homolog b (UNC5b) and vascular endothelial growth factor receptor-2 (VEGFR-2). Additionally, these cells are thought to lack a vascular lumen and minimally proliferate. In contrast, stalk cells form behind tip cells in endothelial sprouts, which proliferate and form vascular lumens^5–7^. These distinct endothelial cell phenotypes highlight the complex dynamics associated with angiogenesis and motivate the development of biomimetic models that facilitate the observation or manipulation of select cell types within intact microvascular networks.

Common bottom-up tissue engineering models attempt to recapitulate the physiological process associated with angiogenesis through the combination of multiple cell types, soluble growth factors, and extracellular matrix proteins. The development of *in vitro* models, including microfluidic devices^8^, two-dimensional^9^ and three-dimensional^10^ culture systems have advanced our understanding of the mechanisms that drive blood vessel formation. However, a gap still exists between our understanding of the behavior of microvascular networks stemming from these bottom-up approaches and physiological microvascular networks. Moreover, the phenotypic and functional physiology associated with blood vessels in common tissue engineered platforms are not typically compared to *in vivo* conditions highlighting both the need and the opportunity to bridge engineering and physiology research perspectives.

Alternative strategies to bottom-up engineering approaches include top-down tissue culture methods, which maintain the complexity of tissues *ex vivo*^11–13^. An example of a top-down tissue culture strategy includes the rat mesentery culture model, developed by our laboratory to investigate cell-cell interactions during angiogenesis in viable, intact microvascular networks with time-lapse capabilities. The rat mesentery culture model takes advantage of whole mount mesentery tissue, providing a simple two-dimensional view of a three-dimensional tissue, down to the single cell level. Previously we have demonstrated that our model can be used to investigate pericyte-endothelial interactions during capillary sprouting^14^, to study smooth muscle constriction^15^, and to evaluate anti-angiogenic drug effects^16^. The innate complexity of the mesentery tissue, which includes blood vessels, lymphatic vessels, interstitial cells, and nerves, combined with the time-lapse capability highlights the advantages of the rat mesentery culture model as an alternative strategy to tissue engineering approaches, which often lack the diversity of cell types and signaling cues that collectively influence endothelial cell behavior during angiogenesis. Still, the critical question remains whether vascular endothelial cells in the rat mesentery culture model display phenotypes similar to *in vivo* endothelial cells during angiogenesis.

The objective of this study was to compare endothelial cell phenotypes along capillary sprouts in cultured *ex vivo* microvascular networks to *in vivo* angiogenic endothelial cells. Herein, we examined the angiogenic marker labeling of endothelial cells from the rat mesentery culture model and compared to *in vivo* endothelial cells stimulated by wound healing angiogenesis. Our results suggest that endothelial cells along capillary sprouts in cultured mesentery microvascular networks 1) maintain an angiogenic phenotype similar to *in vivo* characterized by VEGFR-2 and UNC5b labeling patterns, 2) extend actin-positive pseudopodia at the distal edges, and 3) display a CD36-low phenotype that diminishes along the length of the capillary. Previous work from our laboratory focused on demonstrating the feasibility of stimulating angiogenesis and time-lapse imaging with the rat mesentery culture model^14,16^. The findings from this current study confirm that *ex vivo* cultured endothelial cells in rat mesentery tissue maintain a phenotype similar to *in vivo* endothelial cells and support the physiological relevance of the rat mesentery culture model as a tool to investigate endothelial cell dynamics during angiogenesis within an intact microvascular network. As this type of phenotypic characterization is surprisingly atypical of biomimetic model development, our results offer an important comparative description and suggest a new benchmark for model validation studies.

## Results

### Endothelial Cells Along Capillary Sprouts Display Similar Phenotypes *Ex Vivo* and *In Vivo*

**Figure 1:**
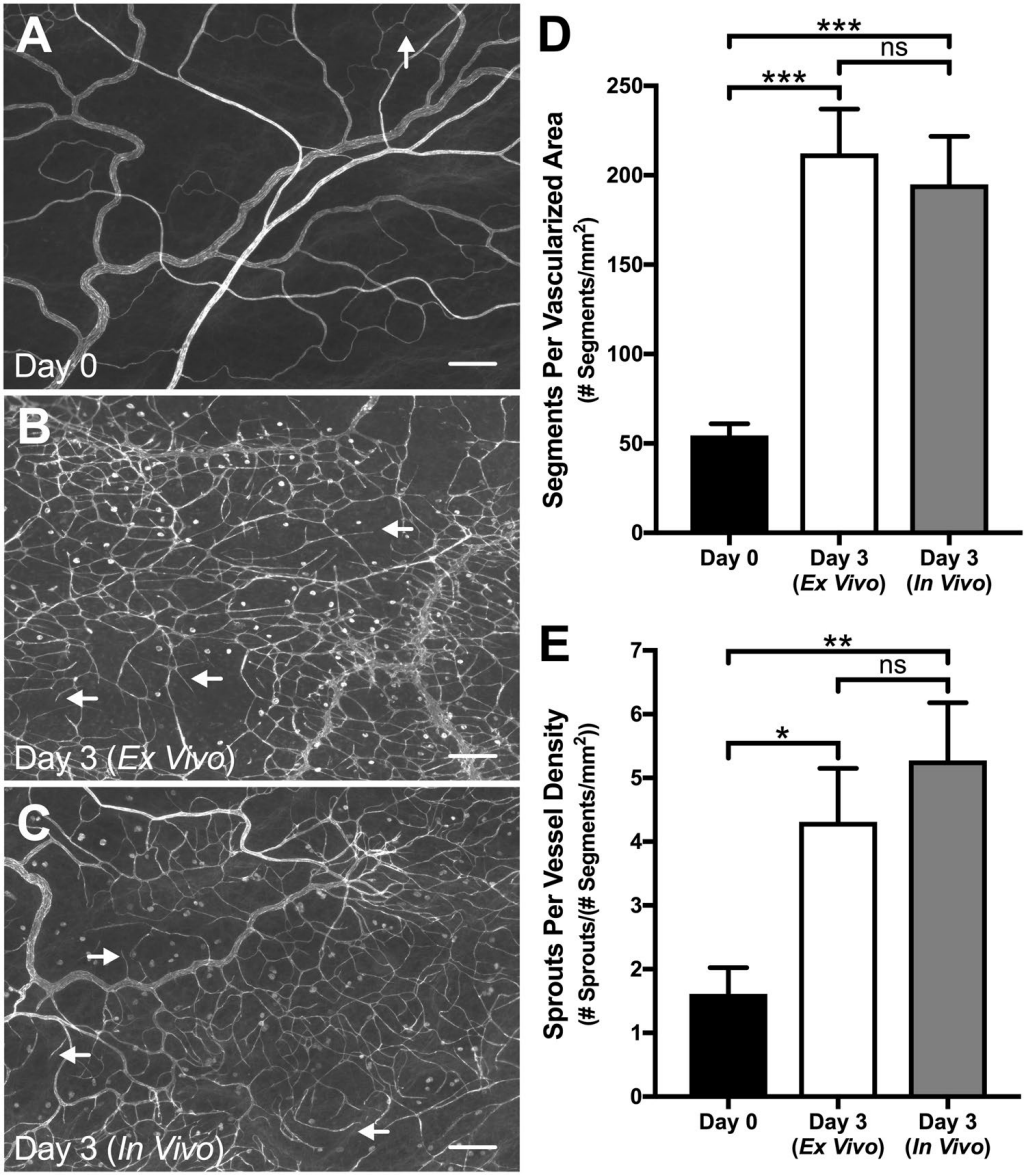
Angiogenesis in *ex vivo* cultured microvascular networks is similar to angiogenic *in vivo* microvasculature. (**A–C**) Comparison between Day 0 (**A**) and both Day 3 (*Ex Vivo*) (**B**) and Day 3 (*In Vivo*) (**C**) groups revealed an increase in capillary sprouting and vessel density. Microvascular networks were visualized with PECAM labeling. Arrows identify examples of capillary sprouts. Scale bars = 200 µm. (**D**,**E**) Vessel density and capillary sprouting were quantified from Day 0 tissues and both Day 3 (*Ex Vivo*) and Day 3 (*In Vivo*) angiogenic tissues. Black, white, and grey bars represent Day 0, Day 3 (*Ex Vivo*), and Day 3 (*In Vivo*) groups respectively. The *^,^ **, and *** indicate a significant difference of p < 0.05, p < 0.01, and p < 0.001, respectively by One-Way ANOVA and Holm-Sidak post hoc method. “ns” indicates no significant difference (p > 0.05).

PECAM labeling of tissues from Day 0 and both Day 3 (*Ex Vivo*) and Day 3 (*In Vivo*) groups identified endothelial cells along blood vessels within the rat mesentery microvasculature (Fig. 1A–C). Cultured *ex vivo* networks and stimulated *in vivo* networks underwent angiogenesis, supported by a significant increase in vessel density from Day 3 (*Ex Vivo*) (212 ± 24.8 segments/vascular area, n = 8; p = 0.0001) and Day 3 (*In Vivo*) (195 ± 26.9 segments/vascular area, n = 8; p = 0.0003) groups compared to the Day 0 group (54.5 ± 6.54 segments/vascular area, n = 8) (Fig. 1D). Comparison of the number of sprouts per vessel density between Day 0 tissues (1.6 ± 0.41 sprouts/vessel density, n = 8) and Day 3 (*Ex Vivo*) (4.3 ± 0.84 sprouts/vessel density, n = 8; p = 0.0376) and Day 3 (*In Vivo*) (5.3 ± 0.91 sprouts/vessel density, n = 8; p = 0.0072) revealed a significant increase for both experimental groups (Fig. 1E). No significant difference was observed in the evaluation of vessel density (p = 0.5740) or endothelial sprouting (p = 0.3751) between the Day 3 (*Ex Vivo*) and Day 3 (*In Vivo*) groups, which suggests that the cultured *ex vivo* networks undergo similar microvascular remodeling as the *in vivo* microvasculature during angiogenesis. Additional *ex vivo* culture experiments were performed in serum-free conditions for the Day 3 (No FBS) control group to evaluate the sham effect of culture conditions (see Supplementary Fig. S1).

**Figure 2:**
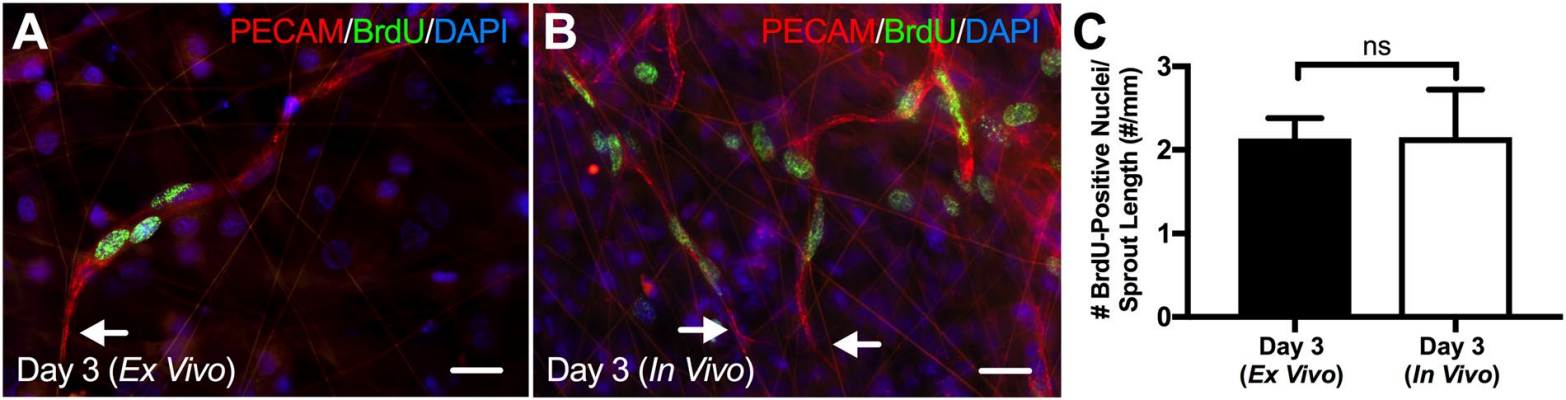
*Ex vivo* endothelial cells proliferate during angiogenesis in the rat mesentery culture model. Qualitative comparison of Day 3 (*Ex Vivo*) (**A**) and Day 3 (*In Vivo*) (**B**) tissues revealed similar proliferation patterns along capillary sprouts. Arrows identify the distal edges of capillary sprouts. Scale bars = 20 µm. (**C**) Quantitative comparison of the number of BrdU-positive nuclei per sprout length for Day 3 (*Ex Vivo*) and Day 3 (*In Vivo*) groups. Black and white bars represent the Day 3 (*Ex Vivo*) and Day 3 (*In Vivo*) group, respectively. “ns” indicates no significant difference (p > 0.05) by Student’s t-test.

To determine whether endothelial cells along sprouts proliferate in the *ex vivo* model of angiogenesis, we performed double labeling of BrdU with PECAM (Fig. 2). We observed endothelial cell proliferation along capillary sprouts in both Day 3 (*Ex Vivo*) (n = 10 tissues, 3 rats) and Day 3 (*In Vivo*) (n = 16 tissues, 2 rats) tissues (Fig. 2A,B). Double immunopositive BrdU and DAPI nuclei were commonly observed along the length of sprouts, including at the most distal nuclei location for both experimental groups. Quantitative analysis of the number of BrdU-positive nuclei along the length of capillary sprouts revealed no significant difference (p = 0.9843) between Day 3 (*Ex Vivo*) (n = 3 tissues, 2 rats) and Day 3 (*In Vivo*) (3 tissues, 2 rats) groups (Fig. 2C).

**Figure 3:**
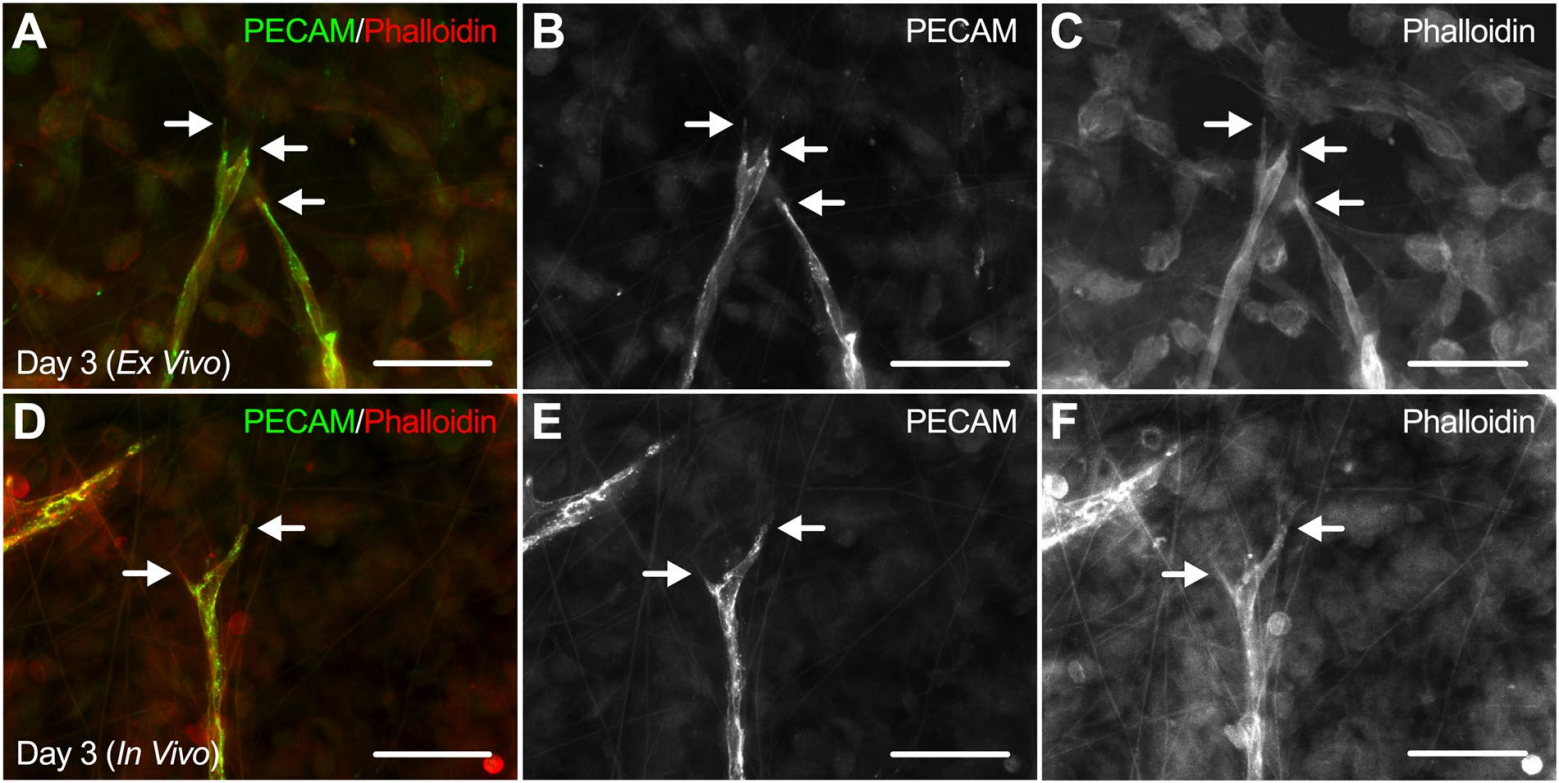
Endothelial cells along *ex vivo* capillary sprouts label positively for actin. Qualitative evaluation of actin labeling between Day 3 (*Ex Vivo*) (**A–C**) and Day 3 (*In Vivo*) (**D–F**) tissues revealed similar labeling patterns in both models of angiogenesis. Arrows identify actin-positive pseudopodia at the distal edges of capillary sprouts. Scale bars = 50 µm.

Actin cytoskeleton remodeling has a primary mechanistic role in endothelial cell migration and pseudopodia extension during capillary sprouting. Here, actin was stained with conjugated phalloidin and PECAM labeling was used to identify endothelial cells along the length of capillary sprouts in both Day 3 (*Ex Vivo*) (n = 6 tissues, 3 rats) and Day 3 (*In Vivo*) (n = 7 tissues, 4 rats) groups (Fig. 3). While examples of long, slender filopodia have been observed in both models of angiogenesis, blunt-ended actin-positive pseudopodia were more commonly seen at the distal edge of leading endothelial cells along capillary sprouts (Fig. 3C,F).

**Figure 4:**
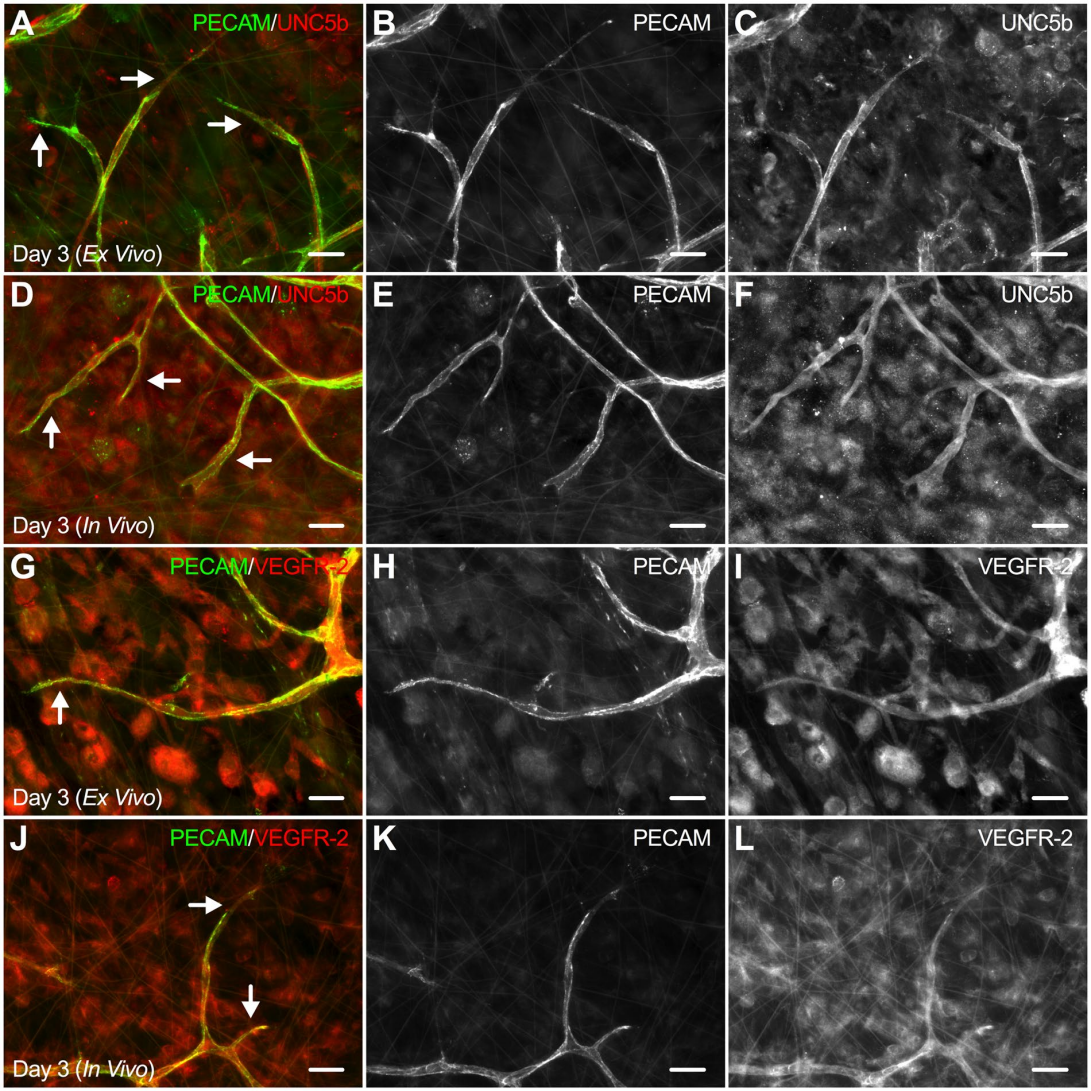
*Ex vivo* capillary sprouts are UNC5b-positive and VEGFR2-positive during angiogenesis in the rat mesentery culture model. Evaluation of UNC5b (**A–F**) and VEGFR-2 (**G–L**) labeling between Day 3 (*Ex Vivo*) and Day 3 (*In Vivo*) tissues revealed positive labeling along the entire length of capillary sprouts in both models of angiogenesis. Arrows identify capillary sprouts. Scale bars = 20 µm.

Leading endothelial cells along sprouts during angiogenesis have been characterized to display positive labeling for UNC5b^17^ and VEGFR-2^4^. These markers were selected for this study because they are commonly found in the literature to identify endothelial cells at the leading edge of a sprout^5–7^. For this study, we performed double labeling of UNC5b or VEGFR-2 and PECAM in Day 3 (*Ex Vivo*) (UNC5b: n = 10 tissues, 5 rats; VEGFR-2: n = 11 tissues, 4 rats) and Day 3 (*In Vivo*) (UNC5b: n = 7 tissues, 3 rats; VEGFR-2: n = 7 tissues, 3 rats) tissues and found endothelial cells were positive for both labels along the length of capillary sprouts (Fig. 4). Interestingly, qualitative observations did not identify intensity labeling differences between leading tip cells along sprouts and more proximal stalk cells for either the *ex vivo* or *in vivo* groups.

**Figure 5:**
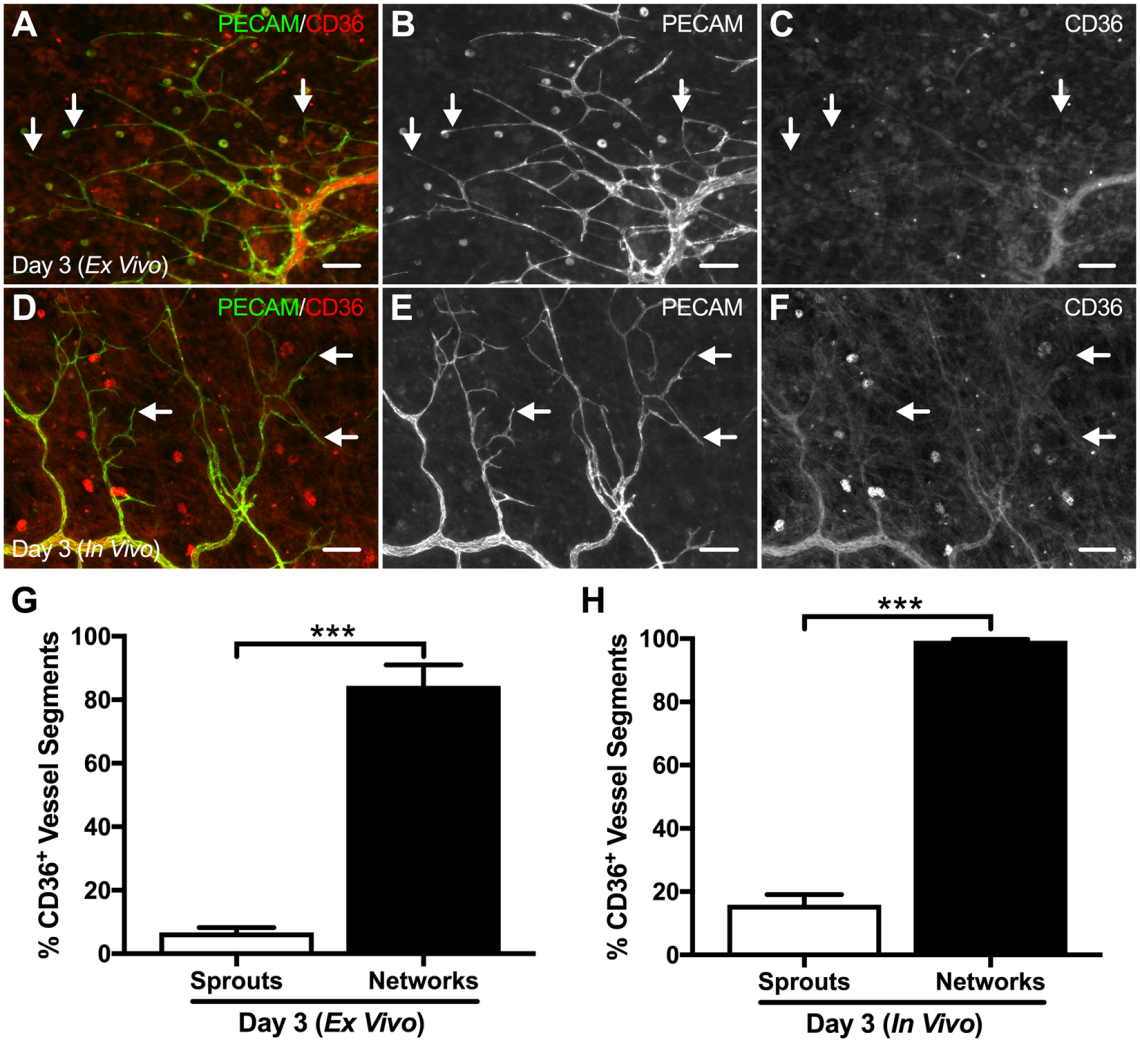
Illustration of CD36 labeling in *ex vivo* cultured microvascular networks and stimulated *in vivo* microvasculature. Comparison between Day 3 (*Ex Vivo*) (**A–C**) and Day 3 (*In Vivo*) (**D–F**) microvascular networks revealed diminishing CD36 labeling along the length of the capillary sprout becoming absent at the tip. Arrows identify examples of CD36-negative capillary sprouts. Scale bars = 200 µm. (**G**,**H**) Capillary sprouts exhibited a substantial decrease in CD36 labeling compared to larger network vessels ( > 10 µm) in Day 3 (*Ex Vivo*) and Day 3 (*In Vivo*) tissues. White and black bars represent “Sprouts” and “Networks” respectively for Day 3 (*Ex Vivo*) and Day 3 (*In Vivo*) groups. The *** indicates a significant difference of p < 0.001 by Mann-Whitney U test.

Another endothelial cell marker characterized in this study was CD36, a thrombospondin-1 receptor^18^. CD36 was selected because it had been previously shown to be down-regulated in capillary sprouts during angiogenesis in rat mesentery tissue stimulated *in vivo* by a chronic wound healing stimulus^19^. Endothelial cells along sprouts in Day 3 (*Ex Vivo*) cultured tissues displayed a similar CD36-low phenotype as previously reported, where CD36 labeling progressively diminished along the microvasculature becoming nearly undetectable at the tip of the sprout (Fig. 5A–F). Quantitative analysis of the percentage of CD36-positive endothelial segments at the capillary level ( < 10 µm) from Day 3 (*Ex Vivo*) tissues revealed a significant decrease in CD36-positive capillary sprouts (6.7 ± 1.5%; n = 8; p = 0.0002) compared to the microvascular network (84 ± 6.6%; n = 8) (Fig. 5G). In line with the previous report, these same CD36 labeling patterns were observed in the Day 3 (*In Vivo*) group, where the percentage of CD36-positive endothelial segments at the capillary level was significantly decreased (16 ± 3.2%; n = 8; p = 0.0002) compared to the microvascular network (99 ± 0.5%; n = 8) (Fig. 5H).

### Capillary Sprouts are Perfused During Angiogenesis *In Vivo*

**Figure 6:**
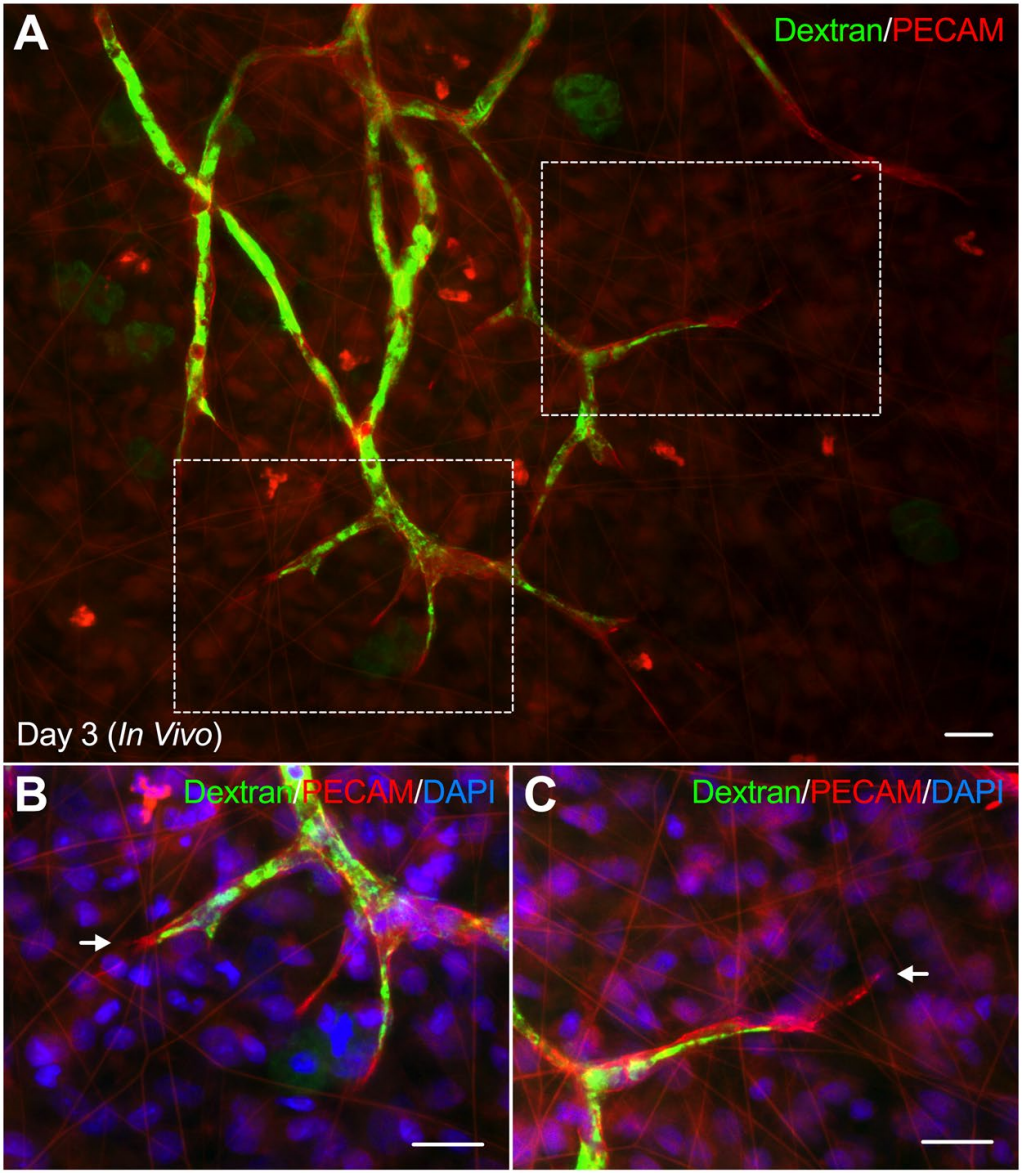
Illustration of *in vivo* vascular lumens during angiogenesis. (**A**) Dextran perfusion of angiogenic *in vivo* microvascular networks identifies vascular lumens in capillary sprouts. (**B**,**C**) Higher magnifications of capillary sprouts in the tissue region, indicated by the above squares. Representative examples highlight the vascular lumen extending to the tip of a capillary sprout (**B**) as well as the vascular lumen forming behind the tip of the sprout (**C**). Arrows identify the distal edges of capillaries. Scale bar = 20 µm.

Vascular perfusion with FITC-conjugated fixable 40 kDa dextran identified patent microvascular networks, including capillaries and sprouts (n = 16 tissues; 2 rats) (Fig. 6). Additional labeling with PECAM visualized endothelial cells and allowed for the investigation of lumenized capillary sprouts. FITC-positive vascular lumens extending to the distal endothelial cell located at the tip along PECAM-positive sprouts (Fig. 6B) as well as non-lumenized sprouts (Fig. 6C) were observed.

## Discussion

The main contribution of this study is the demonstration that angiogenic *ex vivo* endothelial cells in cultured rat mesentery microvascular networks maintain a phenotype similar to angiogenic *in vivo* endothelial cells. To our knowledge, this is the first demonstration of an *ex vivo* model that maintains endothelial cell phenotypes during culture, which is the goal for angiogenesis biomimetic models. These results further establish the rat mesentery culture model as we demonstrate physiological relevance, a primary goal for biomimetic models, in that endothelial cell phenotype dynamics at different locations within a microvascular network are maintained *ex vivo* compared to those observed in native tissues. In addition, our characterization of endothelial cell phenotypes along capillary sprouts in both *ex vivo* and *in vivo* experimental groups suggests the paradigm of tip versus stalk cell identity during angiogenesis is not applicable to every tissue^4,20,21^.

Endothelial cell phenotypes in cultured mesentery tissues were examined by labeling for two common angiogenic markers, VEGFR-2 and UNC5b. VEGFR-2 and UNC5b markers were selected for this study because their positive expression has been well documented in leading endothelial cells of sprouts during angiogenesis^4,17,22^. VEGFR-2 is a dominant mitogenic and chemotactic regulator of endothelial cell responsiveness to vascular endothelial growth factor (VEGF) that positively drives migration^4,23^ and UNC5b is a netrin receptor that negatively regulates endothelial cell sprouting through repulsive cues during capillary branching^17^. Observations of similar labeling patterns for VEGFR-2 and UNC5b in both *ex vivo* and *in vivo* angiogenic tissues suggests endothelial cells cultured in the rat mesentery culture model maintain a similar phenotype to *in vivo* endothelial cells. Interestingly, VEGFR-2 and UNC5b expression has been shown to be upregulated in tip cells versus stalk cells along capillary sprouts when characterized by immunostaining, and have contributed to the tip cell paradigm for endothelial cell phenotypic identities during angiogenesis^5–7^. The majority of the evidence for these labeling patterns have been described for angiogenesis in mouse retina, mouse hindbrain, and zebrafish models of development. In our study, VEGFR-2 and UNC5b labeling along capillary sprouts did not differ between leading tip cells and stalk cells *in vivo* and *ex vivo*. Furthermore, tip cells have been characterized to protrude numerous filopodia extensions during angiogenesis to sense attractive and repulsive cues and migrate in response^6,7^. While filopodia extensions were observed, more common examples of blunt-ended pseudopodia extensions were seen at the distal edge of capillary sprouts for the *in vivo* and *ex vivo* mesentery tissues, consistent with previous reports of capillary growth in the mesentery^24^. Thus, our observations suggest that the common tip cell paradigm might be tissue-specific and more relevant to developmental angiogenesis models than angiogenesis in mature microvascular networks.

The phenotypic similarity between capillary sprouts in the rat mesentery culture model and *in vivo* is further supported by the dynamic downregulation of CD36, a receptor for thrombospondin-1, which is known to be a negative regulator of angiogenesis^18^. In 2008, Anderson *et al*. demonstrated that *in vivo* CD36 receptor labeling is downregulated during inflammation-induced angiogenesis in the rat mesentery after three days post-stimulation. In this study, we observed a similar pattern in cultured *ex vivo* tissues where CD36 labeling diminished along the length of the capillary sprout until becoming nearly absent at the distal edge. Quantitative evaluation of the percent of CD36-positive endothelial segments in the Day 3 (*Ex Vivo*) group revealed a significant decrease (p = 0.0002) between the vascular network and capillary sprouts, thereby confirming our qualitative observations. Similar findings were observed in the Day 3 (*In Vivo*) group as well. Interestingly, there is a significant decrease (p = 0.0217) in the percent of CD36-positive segments from sprouts between the Day 3 (*Ex Vivo*) and Day 3 (*In Vivo*) groups. Possible explanations for this difference include the different angiogenic stimuli for each experimental group, where angiogenesis in *ex vivo* tissues was induced by fetal bovine serum and *in vivo* tissues by wound healing. We hypothesize that these distinct stimuli may elicit differential cellular responses and kinetics of CD36 expression. Also, the work of Anderson *et al*. demonstrates a correlation between sprout length and CD36 labeling, where CD36-negative sprouts were longer (>100 µm) than CD36-positive sprouts^19^, which suggests that the difference observed in CD36 labeling between Day 3 (*Ex Vivo*) and Day 3 (*In Vivo*) groups may be due to sprout length. Additionally, the study demonstrates that CD36 labeling is upregulated in endothelial cells by shear stress *in vitro*, and that CD36 is restored in patent microvascular networks following angiogenic stimuli, which motivates consideration of the role for fluid shear stress in mediating CD36 expression during angiogenesis.

A current limitation of the rat mesentery culture model is the lack of perfusion in the microvasculature and other shear stress-related effects which have been shown to regulate capillary sprouting^25^. However, the absence of flow is not uncommon in other *ex vivo* tissue culture models including the aortic ring^11^ and retinal explant^12^ assays, which have provided key insights into the mechanisms of angiogenesis. Recent advancements in *in vitro* models of capillary sprouting, specifically microfluidic-based models, have proven the importance of flow and shear stress during angiogenesis and has motivated future work of incorporating flow into the rat mesentery culture model^25,26^. A potential approach for achieving flow in the microvascular networks of our model would be cannulation, similar to isolated vessel preparations, of the large artery/vein pair that feed into the mesentery tissue. However, given the number of network outputs and the levels of pressurization needed in upstream arterioles to maintain physiological flow levels throughout a network remain to be investigated. Despite the lack of flow, we have demonstrated that *ex vivo* endothelial cells in our model maintain a phenotype similar to *in vivo* endothelial cells during angiogenesis through comparison of angiogenic markers and proliferation profiles (Figs. 2–4). Interestingly, these findings suggest the lack of flow in the rat mesentery culture model does not affect endothelial cell phenotypes during angiogenesis. Additionally, our observed examples of perfusion to the distal tips of capillary sprouts (Fig. 6B) further challenges the tip cell paradigm that describes tip cells only existing beyond the sprout lumen.

For this study, we used the mesentery exteriorization model to stimulate angiogenesis *in vivo*. While the exact mechanisms of action are unknown, this stimulation has been associated with mast cell activation and an increase of local histamine levels^27^. We selected this *in vivo* angiogenesis model because it produces a robust remodeling response across the hierarchy of microvascular networks over the time course of a few days^15,19,28^. Serum stimulation during culture over a three-day time course was similarly selected based on our previous studies characterizing a similar robust angiogenesis effect^14,16^. In the study by Stapor *et al*., we confirmed that cultured tissues remain viable up to seven days in culture^14^. We have also observed viable cells and network regions with a high density of endothelial cell segments post-fourteen days in culture (data not shown), supporting the ability to culture tissues for longer time periods. Future studies will undoubtedly be needed to confirm that *ex vivo* and *in vivo* endothelial cell phenotype similarities are maintained during different angiogenesis stimuli and longer culture time-points. These studies will be important for identifying the specific triggering mechanisms of angiogenesis for our chosen methods since serum stimulation and mesentery exteriorization remain unknown.

We have previously demonstrated the use of the rat mesentery culture model for both anti-angiogenic and pro-angiogenic studies. Azimi *et al*. confirmed the response of cultured tissues to anti-angiogenic drug treatment by inhibiting angiogenesis using sunitinib and bevacizumab supplementation in the media^16^. Stapor *et al*. demonstrated the use of supplementing media with either basic fibroblast growth factor (bFGF) or VEGF plus platelet-derived growth factor (PDGF) growth factor stimulation as well as fetal bovine serum to induce angiogenesis^14^. In this study, qualitative observations of mesentery tissues harvested and cultured in media supplemented with either bFGF or VEGF plus PDGF for three days revealed UNC5b-positive labeling along the length of capillaries sprouts (Supplementary Figure S2), suggesting that endothelial cell labeling during angiogenesis might be conserved across different stimulation methods. These results combined with our previous characterization of capillary sprouting responses to anti- and pro-angiogenic molecules uniquely validate the rat mesentery culture model as a platform for therapeutic evaluation.

The development of the rat mesentery culture model was motivated by 1) limitations with chronic *in vivo* studies highlighted by the inability to probe specific cellular and molecular interactions and 2) current *in vitro* models that lack physiologically relevant complexity. The rat mesentery culture model provides a novel top-down tissue culture approach for biomimetic tissue engineering strategies that captures the complexity of an *in vivo* tissue in an *ex vivo* platform enabling time-lapse imaging and increased efficiency of tissue-specific drug testing^16,29^. Our model is unique because growth occurs within an intact microvascular network, where angiogenesis can be evaluated at different locations along the hierarchy of the vasculature. A major advantage of the rat mesentery culture model is the capability of time-lapse imaging, where cell-cell interactions can be observed real-time across an intact microvascular network^16^. Other biomimetic models of angiogenesis offer this capability, including microfluidic devices^25^, aortic ring assays^11^, and endothelial tube forming assays^30^. However, since the mesentery tissue is inherently thin (20–40 µm), observation of entire microvascular networks is easily achieved by simple epifluorescent imaging^31^. Our model exploits whole mount mesentery tissue that enables a two-dimensional view of a three-dimensional tissue, down to the single level, across the hierarchy of an intact network. Another advantage of our culture method is its simplicity. In the rat mesentery culture model, tissues can be pinned flat at the bottom of a culture plate using a commercially available polycarbonate filter membrane fitted to an insert, allowing for time-lapse imaging directly in the culture plate. Considering the complexity mismatch between *in vivo* and current *in vitro* methods, our current results of maintained endothelial cell phenotypes during angiogenesis supports the use of the rat mesentery culture model to bridge the gap.

In summary, our results suggest that endothelial cells in the rat mesentery culture model maintain a phenotype similar to *in vivo* endothelial cells during angiogenesis and support the novelty of our model as a biomimetic tool for microvascular research. Moreover, this study highlights the importance of characterizing physiological phenotypes for model development.

## Materials and Methods

### *Ex Vivo* Rat Mesentery Culture Model

All animal experiments were approved by Tulane University’s Institutional Animal and Care Use Committee and performed in accordance with the U.S. Animal Welfare Act, U.S. Public Health Service Policy on the Humane Care and Use of Laboratory Animals, and the NIH *Guide for the Care and Use of Laboratory Animals*. Rat mesentery tissues were harvested and cultured according to our previous descriptions^14,16^. Adult male Wistar rats (325–350 g) were anesthetized via an intramuscular injection of ketamine (80 mg/kg body weight) and xylazine (8 mg/kg body weight). The mesentery was aseptically exteriorized using the ileum as a reference point and the rat was euthanized by an intracardiac injection of 0.2 ml Beuthanasia.

Vascularized mesentery tissues, defined as thin/translucent connective tissue between feeding artery/vein pairs of the small intestine, were aseptically harvested. Tissues were immediately rinsed in sterile phosphate-buffered saline (PBS; Gibco; Grand Island, NY) with CaCl_2_ and MgCl_2_ at 37 °C and immersed in minimum essential media (MEM; Gibco; Grand Island, NY) containing 1% Penicillin-Streptomycin (PenStrep; Gibco; Grand Island, NY). The tissues were then transported to a sterile culture hood to be transferred into 6-well culture plates containing media for each experimental group. Each tissue was spread out on the bottom of a well and secured in place with a thin polycarbonate filter membrane fitted to a cell-crown insert (Sigma-Aldrich; St. Louis, MO). Each well contained one tissue with 4 ml of media with 1% PenStrep and cultured for three days under standard incubation conditions (5% CO_2_, 37 °C) and media was changed every day. To stimulate angiogenesis for the Day 3 (*Ex Vivo*) group, media was supplemented with 10% Fetal Bovine Serum (FBS; Gibco; Grand Island, NY).

### *In Vivo* Wound Healing Angiogenesis

To stimulate angiogenesis for the Day 3 (*In Vivo*) group, an *in vivo* chronic wound healing model was used. Adult male Wistar rats (325–350 g) were anesthetized with an intramuscular injection of ketamine (80 mg/kg body weight) and xylazine (8 mg/kg body weight) followed by a subcutaneous injection of Buprenorphine (0.05 mg/kg body weight). Using aseptic techniques, an incision was made in the abdominal cavity and the mesentery was exteriorized onto a plastic stage for twenty minutes. Exposed tissues were superfused with sterile saline containing 0.9% NaCl (Baxter; Deerfield, IL) at 37 °C and two centrally located vascularized tissues were marked with 7-0 silk sutures (Ethicon; Somerville, NJ). After the 20-minute exteriorization, the mesentery was returned to the abdomen and the incision was sutured using 5-0 and 4-0 silk sutures (Ethicon; Somerville, NJ). The rats were allowed to recover from anesthesia and had access to food and water for the next three days. After three days, the rats were anesthetized and mesentery tissues were again exteriorized. After locating the 7-0 suture marked tissues, the rat was euthanized by an intracardiac injection of 0.2 ml Beuthanasia and vascularized mesentery tissues were harvested. The tissues were immediately rinsed in sterile PBS containing CaCl_2_ and MgCl_2_ and submerged in MEM with 1% PenStrep at 37 °C. Mesentery tissues were fixed following the removal of all angiogenic tissues.

This exteriorization method is based on a previously published model of angiogenesis^28,32,33^ in the rat mesentery that has been linked to mast cell activation and an increase of local histamine levels^27^. This model was selected for this study because it produces a robust remodeling response characterized by an increase in vessel density and capillary sprouting over a short time course.

### Image Acquisition

Fixed immunohistochemistry tissues were imaged using 4X (dry, NA = 0.1), 10X (dry, NA = 0.3), and 20X (oil, NA = 1.4) objectives on an inverted IX70 microscope coupled with a Photometrics CoolSNAP EZ camera.

### Immunohistochemistry

Mesentery tissues were fixed in either 100% methanol at −20 °C for 30 minutes or 4% paraformaldehyde (PFA) at room temperature for 10 minutes followed by three washes in PBS. Tissues that required permeabilization for labeling were washed three times in PBS with 0.1% saponin for 10 minutes each. All antibodies were diluted in antibody buffer solution (PBS, 0.1% saponin, 2% bovine serum albumin, 5% normal goat serum) and non-conjugated primary antibodies were incubated at 4 °C overnight. After three washes in PBS, the tissues were incubated with conjugated secondary antibodies for 1 hour at room temperature followed by another three washes in PBS. The following primary antibodies were used: UNC5b (1:100; Abcam; Cambridge, MA), VEGFR-2 (1:50; Santa Cruz Biotechnology; Dallas, TX), Alexa-568 Phalloidin (1:50; Invitrogen; Carlsbad, CA), CD36 (1:100; Novus Biologicals; Littleton, CO), and PECAM (1:200; BD Pharmingen; San Jose, CA). Secondary antibodies included: Goat Anti-Mouse Alexa-594 Fab Fragments, Goat Anti-Rabbit Alexa-594, and Streptavidin Cy-2 (Jackson Immunoresearch Laboratories; West Grove, PA). Nuclear staining was achieved by a 10-minute incubation with DAPI (1:3000; Invitrogen; Carlsbad, CA) at room temperature.

For Day 3 (*In Vivo*) BrdU labeling, a 30 ml BrdU solution (1 mg BrdU per 1 ml sterile saline) was warmed to 37 °C and perfused into the abdominal cavity of an anesthetized rat and incubated for 2 hours. Following incubation, the rat was euthanized and angiogenic tissues were identified by 7-0 suture demarcation, harvested, and fixed in 100% methanol for 30 minutes at −20 °C. BrdU labeling of Day 3 (*Ex Vivo*) tissues was achieved by a 2-hour incubation with BrdU supplemented media (1 mg BrdU per 1 ml MEM + 1% PenStrep) at 37 °C followed by a 30-minute methanol fixation. For both models of angiogenesis, following fixation mesentery tissues were washed three times in PBS, incubated for 1 hour in 2 M HCl, washed three times in PBS + 0.1% saponin, and incubated with anti-BrdU (1:100; Sigma-Aldrich; St. Louis, MO) at 4 °C overnight. Tissues were subsequently labeled for PECAM and DAPI following BrdU labeling.

Vascular lumina were identified in Day 3 (*In Vivo*) tissues by a bolus injection of 2.5 ml lysine fixable 40 kDa FITC-dextran (10 mg per 1 ml sterile saline; Invitrogen; Carlsbad, CA) via the femoral vein of an anesthetized rat. Following injection, the rat was euthanized and mesentery tissues were fixed abdominally in 4% PFA for 10 minutes and harvested for labeling with PECAM and DAPI.

### Quantification of Angiogenesis

Vessel density and capillary sprouting were quantified from one microvascular network per tissue from 10X images for each group. A microvascular network was defined as having a feeding arteriole and draining venule with a branching capillary plexus. Vessel density was defined as the number of vessel segments per vascularized area. Vessel segments were defined as PECAM-positive endothelial cell segments between two branch points. Capillary sprouts were defined as blind-ended PECAM-positive endothelial cell segments originating from a vessel. Quantification of angiogenesis was analyzed for the following groups: 1) Day 0: n = 8 tissues from 4 rats, 2) Day 3 (*Ex Vivo*): n = 8 tissues from 5 rats, and 3) Day 3 (*In Vivo*): n = 8 tissues from 4 rats. Analysis was performed using the NIH Fiji open-source software version 2.0.0^34^, where vessel segments and capillary sprouts were counted using the Cell Counter plugin.

### Quantification of CD36 Labeling

CD36 labeling was quantified as the percent of CD36-positive endothelial cell segments along the “Sprouts” and larger “Network” vessels from one microvascular network per tissue from 10X images for each group. A microvascular network was defined as having a feeding arteriole and draining venule with a branching capillary plexus. The “Sprouts” were identified as blind-ended PECAM-positive endothelial cell segments originating from a vessel and the “Network” was identified as PECAM-positive endothelial cell segments between two branch points of a vessel larger than 10 µm. Observable CD36-positive labeling was visually assessed by comparison to the negative background, similar to previous published descriptions of CD36 analysis^19^. CD36 labeling was analyzed for the following groups: 1) Day 3 (*Ex Vivo*): n = 8 tissues from 4 rats and 2) Day 3 (*In Vivo*): n = 8 tissues from 4 rats. Analysis was performed using the NIH Fiji open-source software version 2.0.0^34^, where “Sprouts” and “Network” were counted using the Cell Counter plugin.

### Statistical Analysis

Data are presented as mean ± standard error of mean (SEM). One-way Analysis of Variance (ANOVA) followed by pairwise comparisons with the Holm-Sidak post hoc test was used to compare vessel density and the number of endothelial sprouts per vessel density between Day 0, Day 3 (*Ex Vivo*), and Day 3 (*In Vivo*) groups. Mann-Whitney rank sum tests were used to compare the percentage of CD36-positive endothelial segments for “Sprouts” and “Networks” in the Day 3 (*Ex Vivo*) and Day 3 (*In Vivo*) groups. Student’s t-test was used to compare the number of BrdU-positive nuclei per sprout length between the Day 3 (*Ex Vivo*) and Day 3 (*In Vivo*) groups. For all tests, a p-value < 0.05 was considered statistically significant. Statistical analysis was performed using GraphPad Prism version 7.00 software.

### Data availability

Raw data includes images and spreadsheets with vessel counts, areas, capillary sprouting, and the number of BrdU-positive nuclei per capillary length per analysis. These datasets will be made available upon request to the corresponding author.

## Electronic supplementary material

Supplementary Information
