# Supplementary material for: Endothelial Cell Phenotypes are Maintained During Angiogenesis in Cultured Microvascular Networks
Source: Sci Rep. 2018 Apr 12;8:5887. doi: 10.1038/s41598-018-24081-z (PMC5897326; doi:10.1038/s41598-018-24081-z)
Supplement: Supplementary file 1 — Supplementary Information [file 41598_2018_24081_MOESM1_ESM.docx]

**Endothelial Cell Phenotypes are Maintained During Angiogenesis in Cultured Microvascular Networks**

Jessica M. Motherwell^1^, Christopher R. Anderson^2^ and Walter L. Murfee^3*^

^1^Department of Biomedical Engineering, Tulane University, New Orleans, LA, 70118, United States

^2^Department of Chemical and Biomolecular Engineering, Lafayette College, Easton, PA, 18042, United States

^3^Department of Biomedical Engineering, University of Florida, Gainesville, FL, 32611, United States

**Materials and Methods**

**Rat Mesentery Culture Model**

All animal experiments were approved by Tulane University’s Institutional Animal and Care Use Committee and performed in accordance with the U.S. Animal Welfare Act, U.S. Public Health Service Policy on the Humane Care and Use of Laboratory Animals, and the NIH *Guide for the Care and Use of Laboratory Animals*. Adult male Wistar rats (325–350 g) were anesthetized via an intramuscular injection of ketamine (80 mg/kg body weight) and xylazine (8 mg/kg body weight). The mesentery was aseptically exteriorized and the rat was euthanized by an intracardiac injection of 0.2 ml Beuthanasia. Vascularized mesentery tissues were harvested and immediately rinsed in sterile PBS with CaCl_2_ and MgCl_2_ at 37 °C and immersed in MEM containing 1% PenStrep. The tissues were then transported to a sterile culture hood to be transferred into 6-well culture plates containing media for each experimental group. Each tissue was spread out on the bottom of a well and secured in place with a thin polycarbonate filter membrane fitted to a cell-crown insert. Each well contained one tissue with 4 ml of media with 1% PenStrep and cultured for three days under standard incubation conditions (5% CO_2_, 37 °C) and media was changed every day.

***Ex Vivo* Angiogenesis Control Study**

For the *ex vivo* angiogenesis control group, tissues were harvested and prepared according to the rat mesentery culture model method described above. One tissue was placed into each well with 4 ml of MEM and 1% PenStrep without serum supplementation according to the following experimental group: 1) Day 3 (No FBS): n = 8 tissues from 2 rats.

***Ex Vivo* Growth Factor Study**

To stimulate angiogenesis, media was supplemented with 200 ng/ml recombinant human basic fibroblast growth factor (bFGF) or 200 ng/ml vascular endothelial growth factor (VEGF) and platelet-derived growth factor (PDGF). Tissues were harvested and prepared according to the rat mesentery culture model method described above. One tissue was placed into each well with 4 ml of MEM with 1% PenStrep and growth factor according to the following experimental groups: 1) Day 3 (bFGF): n = 2 tissues from 1 rat and 2) Day 3 (VEGF/PDGF): n = 2 tissues from 1 rat.

**Immunohistochemistry**

UNC5b/Lectin: Tissues were spread on microscope slides and fixed in methanol at -20 °C for thirty minutes and labeled according to the following protocol: 1) 1:100 UNC5b (Abcam; Cambridge, MA); 2) 1:100 Alexa Fluor 594-conjugated goat anti-rabbit secondary antibody (Jackson ImmunoResearch Laboratories; West Grove, PA) and 1:40 BSI-Lectin conjugated to FITC (Sigma-Aldrich; St. Louis, MO). All antibodies were diluted in antibody buffer solution (PBS, 0.1% saponin, 2% bovine serum albumin, and 5% normal goat serum).

**Quantification of Angiogenesis**

Vessel density and capillary sprouting were quantified from one microvascular network per tissue from 10X images for each group. A microvascular network was defined as having a feeding arteriole and draining venule with a branching capillary plexus. Vessel density was defined as the number of vessel segments per vascularized area. Vessel segments were defined as PECAM-positive endothelial cell segments between two branch points. Capillary sprouts were defined as blind-ended PECAM-positive endothelial cell segments originating from a vessel. Quantification of angiogenesis was analyzed for the following groups: 1) Day 0: n = 8 tissues from 4 rats, 2) Day 3 (10% FBS): n = 8 tissues from 5 rats, and 3) Day 3 (No FBS): n = 8 tissues from 2 rats. Analysis was performed using the NIH Fiji open-source software version 2.0.0^35^, where vessel segments and capillary sprouts were counted using the Cell Counter plugin.

**Statistical analysis**

Data are presented as mean ± standard error of mean (SEM). A one-way Analysis of Variance (ANOVA) followed by pairwise comparisons with the Holm-Sidak post-hoc method was used to analyze the vessel density and the number of endothelial sprouts per vessel density between Day 0, Day 3 (10% FBS), and Day 3 (No FBS) groups. For all tests, a p-value < 0.05 was considered statistically significant. Statistical analysis was performed using GraphPad Prism version 7.00 software.


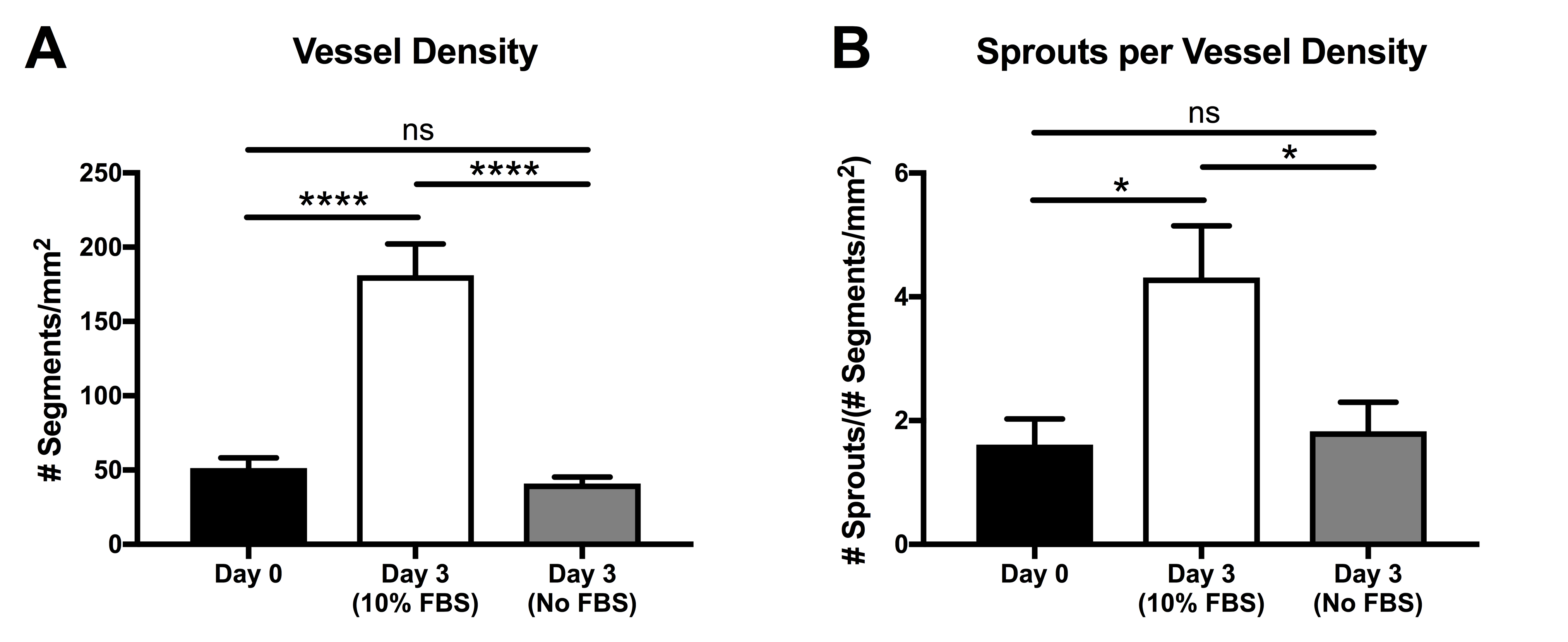


**Supplementary Figure S1. Fetal bovine serum stimulation causes robust angiogenesis in the rat mesentery culture model.** (**A,B**) Comparison between Day 3 (10% FBS) and both Day 3 (No FBS) and Day 0 groups revealed a significant increase in vessel density (**A**) and capillary sprouting (**B**). Black, white, and grey bars represent Day 0, Day 3 (10% FBS), and Day 3 (No FBS) groups respectively. * and **** indicates a significant difference of p < 0.05 and p < 0.0001 by One-Way ANOVA and Holm-Sidak post hoc method. “ns” indicates no significant difference (p > 0.05).


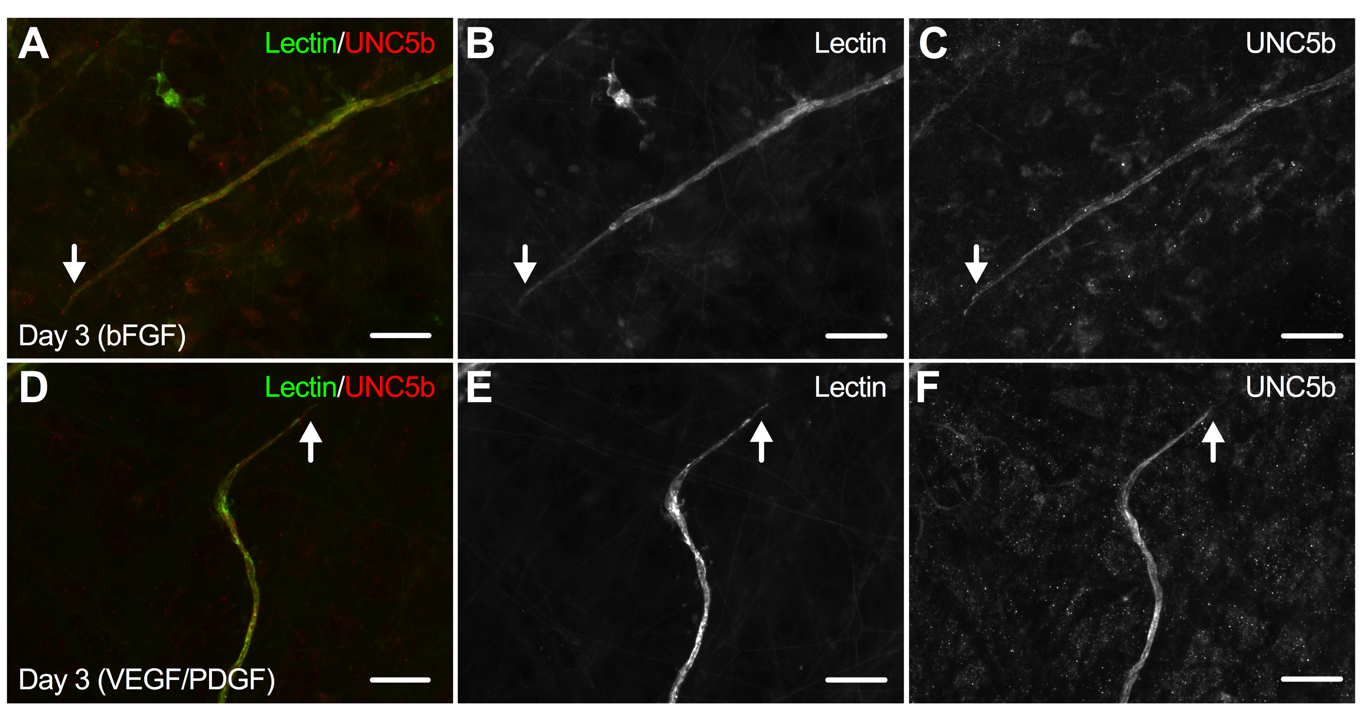


**Supplementary Figure S2. *Ex vivo* capillary sprouts are UNC5b^+^ during growth factor stimulation in the rat mesentery culture model.** Evaluation of UNC5b labeling between Day 3 (bFGF) (**A–C**) and Day 3 (VEGF/PDGF) (**D–F**) stimulated tissues revealed positive labeling along the entire length of capillary sprouts in both models of angiogenesis. Arrows indicate tips of capillary sprouts. Scale bars = 20 µm.
